# Correction to: A cross-validation-based approach for delimiting reliable home range estimates

**DOI:** 10.1186/s40462-017-0116-y

**Published:** 2017-12-05

**Authors:** Eric R. Dougherty, Colin J. Carlson, Jason K. Blackburn, Wayne M. Getz

**Affiliations:** 1 Department of Environmental Science, Policy, and Management, University of California, Berkeley, Berkeley, CA USA; 2 Spatial Epidemiology and Ecology Research Laboratory, Department of Geography, University of Florida, Gainesville, FL USA; 3 Emerging Pathogens Institute, University of Florida, Gainesville, FL USA; 4 School of Mathematical Sciences, University of KwaZulu-Natal, Durban, South Africa

## Correction to: Movement Ecology (2017) 5:19 DOI:10.1186/s40462-017-0110-4

### Original text

A grid-based exploration of parameter space was then conducted (Figure 2), whereby each of the 100 training/testing datasets was analyzed at every combination of *k* and *s* values on the grid. This analysis entailed the creation of local convex hulls with *k* nearest neighbors and a scaling factor of *s*. In all subsequent analyses, we assume that the scaling of time follows a linear formulation; however, when movement patterns more closely exemplify diffusion dynamics, an alternative equation for the TSD may be more accurate [1]. The test points were then laid upon the resulting hulls, and the probability of each was calculated as the proportion of the total number of hulls (equivalent to the total number of points in the training dataset) that contained the test point (Figure 1). Test points that were not contained within any hulls were assigned a probability equal to the inverse of the total number of points in the full movement path divided by 100, effectively penalizing any hull sets that did not include each of the test points. Though an arbitrary selection, the choice of a consistent penalty term across individuals will serve to standardize the procedure. A larger penalty will likely result in a higher optimal *k* value and bear a closer resemblance to the MCP. The natural log of the probability was calculated and information criterion values analogous to Akaike’s Information Criterion (AIC) were derived using the equation:

$$\text{IC} = -2\ * \ \ln\! \:\left(\sum_{i=1}^{n}\! \:P\left(\! \text{test\ points} \ | \ \text{training\ hullsets}\right)\!\right)\: \! + 2\ * \ {k} $$

The choice of 2*k* as the penalty term was made to maintain a structure analogous to the AIC equation. Given the expansive literature concerning the performance and behavior of AIC under various scenarios, maintaining this structure may offer insight into similar strengths and weaknesses of the proposed approach. Ultimately, without such a penalty, all movement paths would tend towards a *k* equal to the number of points in the training set, such that each individual point was assigned a probability of one. It should be noted that this penalty term is specific to the *k* (nearest neighbors) method, but the underlying cross-validation procedure could very easily be extended for the optimization of the *a* (adaptive parameter) method if an appropriate penalty term is selected. An ideal penalty term would likely result in a increase of the information criterion value by a similar magnitude as in the *k*-based formulation above (i.e., ranging from approximately 10^0^ to 10^2^).

### Revised text

A grid-based exploration of parameter space was then conducted (Figure 2), whereby each of the 100 training/testing datasets was analyzed at every combination of *k* and *s* values on the grid. This analysis entailed the creation of local convex hulls with *k* nearest neighbors and a scaling factor of *s*. In all subsequent analyses, we assume that the scaling of time follows a linear formulation; however, when movement patterns more closely exemplify diffusion dynamics, an alternative equation for the TSD may be more accurate [1]. The test points were then laid upon the resulting hulls, and the probability of each was calculated as the proportion of the total number of hulls (equivalent to the total number of points in the training dataset) that contained the test point (Figure 1). Test points that were not contained within any hulls were assigned a probability equal to the inverse of the total number of points in the full movement path divided by 100, effectively penalizing any hull sets that did not include each of the test points. Though an arbitrary selection, the choice of a consistent penalty term across individuals will serve to standardize the procedure. A larger penalty will likely result in a higher optimal *k* value and bear a closer resemblance to the MCP. The natural log of the probability was calculated and information criterion values analogous to the **Bayesian** Information Criterion **(BIC)** were derived using the equation:

$${\begin{aligned} \text{IC} &= -2 * \ln\:\left(\sum_{i=1}^{n} \:P\left(\text{test\ points} \ | \ \text{training\ hullsets}\right)\right)\\ &\quad+ {k}*\ln(P)\\ \end{aligned}} $$

$$\text{where} \ \ P = \sum_{i=1}^{n} \:(\text{test\ points}) $$

The choice of ***k*** **∗ ln(** ***P*** **) as the overall penalty term** was made to maintain a structure analogous to the **BIC** equation. Given the expansive literature concerning the performance and behavior of **BIC** under various scenarios, maintaining this structure may offer insight into similar strengths and weaknesses of the proposed approach. Ultimately, without such a penalty, all movement paths would tend towards a *k* equal to the number of points in the training set, such that each individual point was assigned a probability of one. **An alternative method akin to Akaike’s Information Criterion can also be applied, but the penalty term (2∗** ***k*** **) does not scale with the total number of test points (in turn, a function of the total length of the movement path) and will likely result in higher optimal** ***k*** **values than the BIC analogue.** It should also be noted that this penalty term is specific to the *k* (nearest neighbors) method, but the underlying cross-validation procedure could very easily be extended for the optimization of the *a* (adaptive parameter) method if an appropriate penalty term is selected. An ideal penalty term would likely result in a increase of the information criterion value by a similar magnitude as in the *k*-based formulation above (i.e., ranging from approximately 10^0^ to **10** ^**3**^).

### Explanation of correction

After the publication of this article [2], it came to our attention that the results presented throughout were based on an alternative Information Criterion (IC) equation that did not appear in the original article. The alternate formulation (akin to the Bayesian Information Criterion, rather than Akaike’s Information Criterion) should be calculated as:

$$\begin{aligned} \text{IC} &= -2 * \ln\:\left(\sum_{i=1}^{n} \:P\left(\text{test\ points} \ | \ \text{training\ hullsets}\right)\right)\\ &\quad+ {k}*\ln(P) \end{aligned} $$

$$\text{where} \ \ P = \sum_{i=1}^{n} \:(\text{test\ points}) $$

The only difference between the equation here and the one in the original article is the penalty term. In the equation above, increases in the *k* value are penalized more heavily than the simpler 2∗*k* term. The additional benefit of this equation, and the primary reason for its use in the analysis in [2], is that the penalty term scales (in a non-linear fashion) with the total number of test points, offering more flexibility when considering trajectories of varying lengths.

Despite this issue, the fundamental principles underlying the cross-validation method remain sound, and both the original IC equation and the one presented here can be used with confidence. The logic for utilizing a BIC analogue is the same as that for formulating an AIC analogue; the correction outlined here simply enables the replication of the results in the article. The equation and the text in bold above have been altered from the original version of the paper.
